# Prenatal Diagnosis of Neu–Laxova Syndrome

**DOI:** 10.3390/diagnostics12071535

**Published:** 2022-06-23

**Authors:** Adriana Serrano Olave, Alba Padín López, María Martín Cruz, Susana Monís Rodríguez, Isidoro Narbona Arias, Jesús S. Jiménez López

**Affiliations:** 1 Obstetrics and Gynecology, Materno-Infantil Hospital Regional Universitary Málaga, Avenue Arroyo de los Ángeles S/N, 29011 Málaga, Spain; appadinlopez@gmail.com (A.P.L.); martincruzmaria@gmail.com (M.M.C.); monissusana@gmail.com (S.M.R.); dr.narbona@gmail.com (I.N.A.); jesuss.jimenez.sspa@juntadeandalucia.es (J.S.J.L.); 2 Department of Surgical Specialties, University of Malaga, 29010 Málaga, Spain

**Keywords:** Neu–Laxova syndrome, ultrasound findings, fetal edema, proptosis, intrauterine growth restriction, restrictive dermopathy, facial dysmorphism, genetic study, amniotic fluid

## Abstract

Neu–Laxova syndrome is a rare and lethal genetic disease with autosomal recessive inheritance involving abnormalities of multiple systems. It was first reported in 1971. Since then, just eighty-eight cases have been reported. The syndrome is characterized by early and severe growth restriction, and craniofacial anomalies, such as microcephaly, hypertelorism and other malformations, resulting in quite characteristic features. Additionally, it might appear as generalized edema, flexion contractures and other malformations of the extremities, abnormalities in the CNS (central nervous system), skin (severe ichthyosis), and genitourinary and cardiac abnormalities. We present the case of a patient who had her first pregnancy with a fetus with Neu–Laxova syndrome diagnosed in our center during the second-trimester ultrasound. The ultrasound findings suggested the diagnosis, which was confirmed with a genetic study of the amniotic fluid: the variant of the *PSAT1 gene*, associated with NLS (Neu–Laxova syndrome) 2 in homozygosis. Moreover, there was a second pregnancy with a fetus carrying the same mutation in heterozygosis. In addition, we have carried out a review of published literature about this disease up to the present time.

## 1. Introduction

Neu–Laxova syndrome (NLS) is a lethal disorder with multiple abnormalities. This pathology was described for the first time in 1971 by Neu and in 1972 by Laxova and individualized under this entity that bears the names of these two authors by Lazjuk et al. [1,2,3]. The main manifestations of this syndrome have been summarized under the term “neuro-oculo-ectodermal dysplasia”. These manifestations include intrauterine growth restriction (IUGR), and facial dysmorphism with very characteristic features, such as proptosis, ichthyosis and central nervous system (CNS) malformations dominated by microlissencephaly. Other alterations are hypokinesia, arthrogryposis, subcutaneous edema and pulmonary hypoplasia. Affected children usually die in utero or shortly after birth due to respiratory failure and/or infectious and neurological complications [4]. Eighty-eight cases have been reported to date [5].

**Figure 1:**
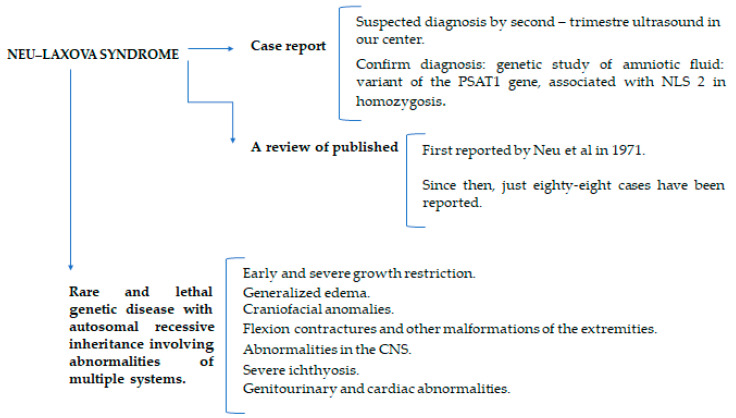
Summary of the main aspects discussed in the manuscript.

Until recently, the etiopathogenesis was unknown. In 2014, a gene mapping study in three consanguineous families affected by NLS identified a mutation in the *PHGDH gene.* This entity was named Neu Laxova 1 syndrome (NLS-1). During the same year, two additional modifiers in genes encoding phosphoserine aminotransferase 1 *(PSAT1)* and phosphoserine phosphatase *(PSPH)* were identified by Acuña-Hidalgo et al. NLS caused by the mutation in the *PSAT1 gene* was classified as Neu Laxova 2 (NLS-2), with a phenotype very similar to 1. These genes are involved in the serine synthesis pathway, vital for the synthesis of brain lipids [6,7]. Therefore, NLS is genetically heterogeneous and can be caused by mutations in the three previously described genes that encode enzymes of the L-serine biosynthetic pathway, an amino acid required for the synthesis of brain lipids sphingolipids and gangliosides. It has an autosomal recessive inheritance pattern and most of the time it is related to consanguinity [3]. This suggests that NLS represents a more severe form within a continuous spectrum of serine-deficiency disorders, and NLS itself might also have a variable clinical expression [7]. Among other things, this is the reason why the prenatal diagnosis of NLS still remains a challenge. The following image (Figure 1) summarizes what was previously stated.

## 2. Materials and Methods

This is a review of all the literature published to date. In total, 81 cases have been published, the last one in 2016. Subsequently, seven more cases have been reported from different laboratories around the world. Therefore, we present a retrospective descriptive cross-sectional study using all the available bibliographies in the usual databases, such as Cochrane, Scopus, Science Direct, or PubMed. In addition, we present two cases that happened in our center in the year 2021, in the Materno-Infantil Universitary Regional Hospital of Malaga. Of course, with the approval of the Hospital Ethics Committee. In the diagnosis of the key case, the collaboration of the Genetics and Prenatal Diagnosis Services had been essential. The objective of this review is to clarify and emphasize the ultrasound characteristics of this rare syndrome as much as possible so that it can be suspected in daily clinical practice and confirmed by appropriate genetic tests which will be later described in the discussion. Ideally, an early gestational age diagnosis offers better advice to family members on the approach and management of this syndrome, which is so complex and has such a poor prognosis.

## 3. Results: Case Presentation

A 26-year-old primigravida patient with no personal or family history of interest. Both parents are healthy, without apparent consanguinity between them. A normal evolution of pregnancy to date, normal first-trimester ultrasound and combined screening for chromosomal abnormalities in the first-trimester: low risk for Down, Edward’s and Patau syndromes. Blood tests of first and second-trimesters without pathological findings. She was assessed by the Prenatal Diagnosis Unit to perform a morphological ultrasound at week 21. The ultrasound examination revealed biometry not in accordance with gestational age, which meant an early diagnosis of severe IUGR (according to 17 + 4 weeks, EFW: 208 g). In addition, a fetal morphological study showed some morphological abnormalities.

**Figure 2:**
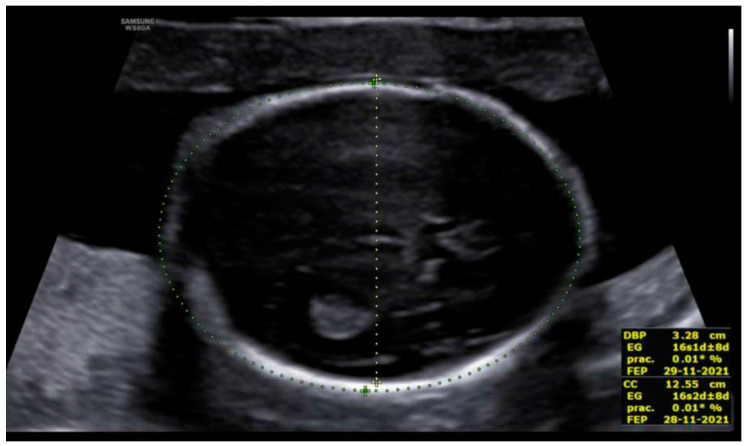
Axial view of midline with non-visualization of the CSP, showing directly the columns of the fornix. Additionally, we can observe microcephaly. Measurements corresponding to a gestational age of 16 weeks.

**Figure 3:**
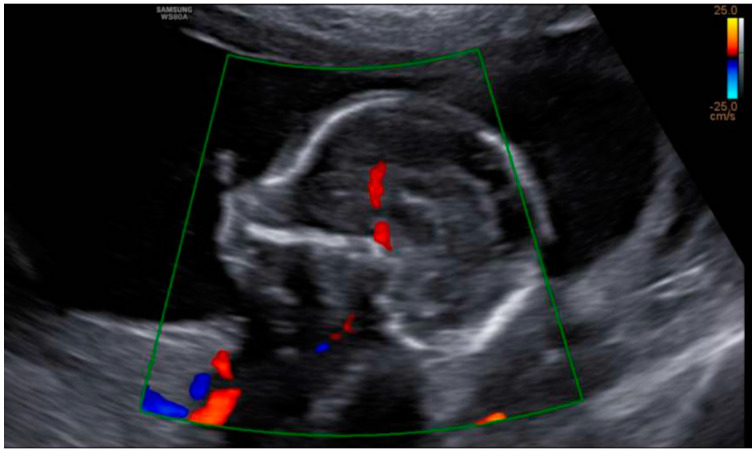
In sagittal section, the corpus callosum is not present. Both findings are consistent with total ACC.

**Figure 4:**
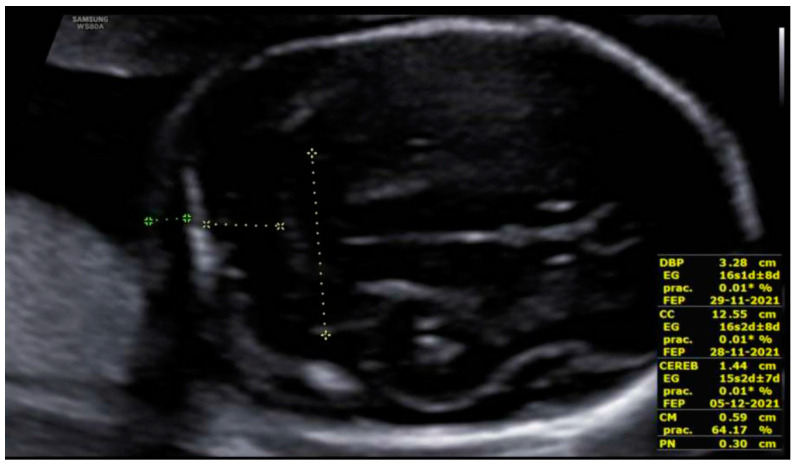
Axial view of the posterior fossa. Hypoplastic cerebellum: it shows cerebellum with a maximum transverse diameter of 14 mm. Cerebellomedullary cistern and nuchal fold within normal range.

At the intracranial level, the cavum septum pellucidum (CSP) was absent and there was an abnormal bridge of tissue across the midline at the level of the CSP, visualizing directly the columns of the fornix. On the prefrontal area, the midline was open in three lines in the sagittal section with an elevation of the third ventricle. Additionally, the corpus callosum was not identified in the sagittal section (Figure 2 and Figure 3). All these indirect findings were suggestive of agenesis of the corpus callosum (ACC). In the posterior fossa, the hypoplastic cerebellum: the cerebellum presented a transverse diameter of 14 mm, according to 15 + 2 weeks and thin thickness (Figure 4). Ventriculomegaly was not observed.

**Figure 5:**
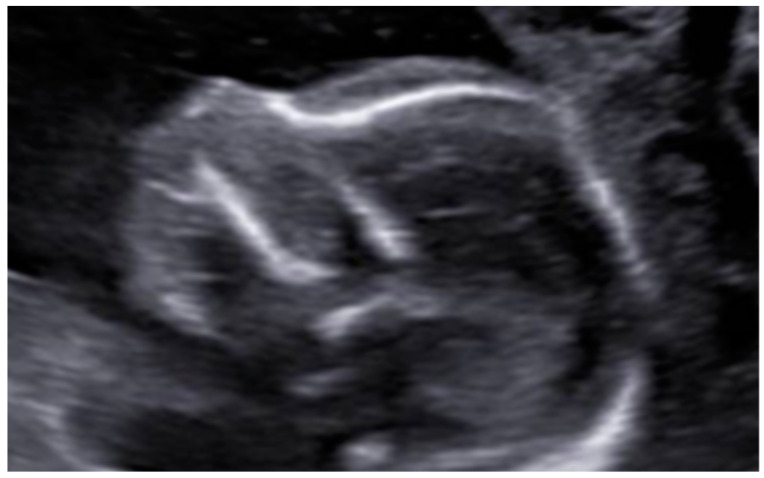
Sagittal section of the fetal profile, where nasal bone, prefrontal edema and mild micrognathia could be seen.

**Figure 6:**
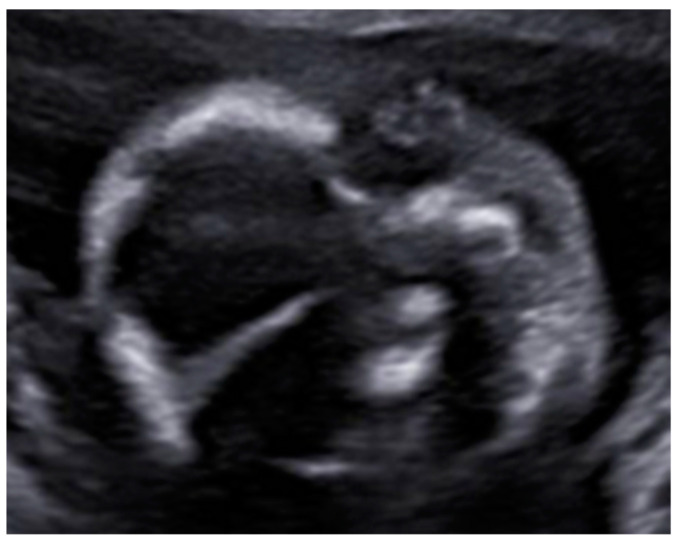
Initial ocular proptosis bilaterally.

**Figure 7:**
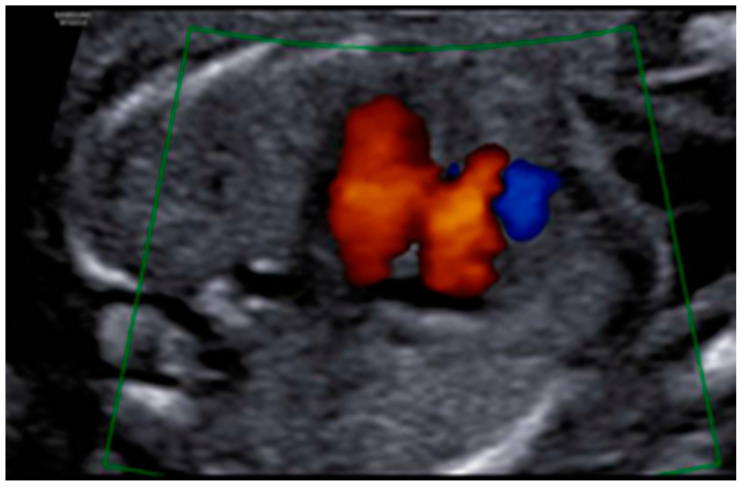
Normal four-chamber Yagel slice.

**Figure 8:**
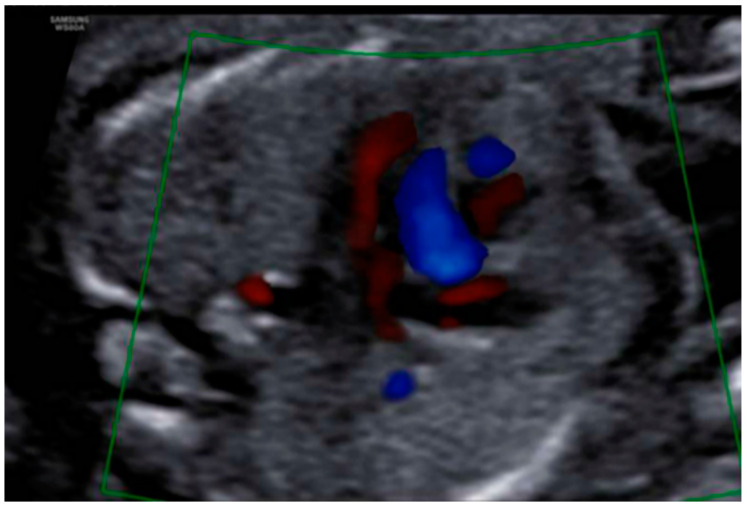
Mild deviation of the cardiac axis to the left.

**Figure 9:**
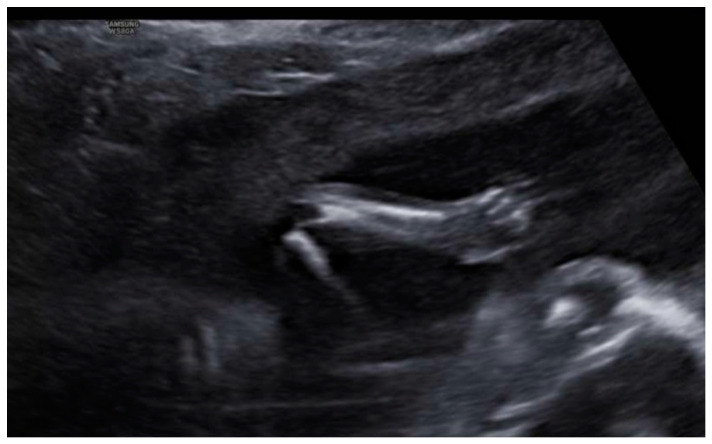
Wrist joint seemed fixed and feet located in hyperflexion with little mobility.

**Figure 10:**
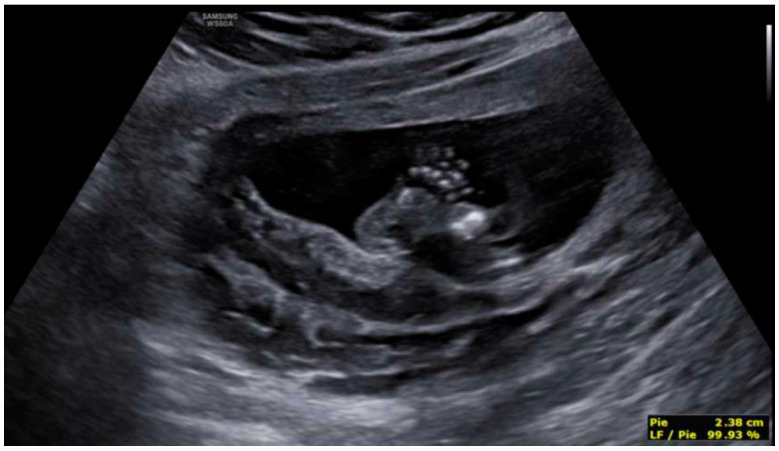
Feet located in forced hyperflexion and with reduced mobility.

Profile with the nasal bone present, although a slightly flattened frontal area was appreciated, with prefrontal edema and subjective micrognathia (Figure 5). Mild ocular proptosis bilaterally (Figure 6). Cardiac study within normal limits, except for slight levocardia (Figure 7 and Figure 8). Vertebral column without apparent anomalies. Normal male genitalia. Regarding the extremities, the fetus presented hands with normal fingers and phalanges, as well as preserved mobility. However, the wrist joint seemed fixed and the feet were located in hyperflexion with little mobility. Feet were located in forced hyperflexion and with reduced mobility (Figure 9 and Figure 10). Nonamniotic fluid alterations were found.

After those described fetal abnormalities, the patient was informed of them and about the fetal prognosis, as well as the possible causes (mainly genetic and infectious). She was offered the possibility of performing an invasive technique to refine the diagnosis and be able to name the syndrome. She accepted and amniocentesis was performed at 21 weeks. Quantitative Fluorescence-Polymerase Chain Reaction (QF-PCR) and comparative genomic hybridization arrays (CGH-arrays) studies were requested from the Genetics Service, cytomegalovirus (CMV) and toxoplasma PCR from the Microbiology Unit and toxoplasmosis, rubella cytomegalovirus, herpes simplex (TORCH virus) serology from the Clinical Analysis Laboratory. Results were as follows: QF-PCR Diploid 13, 18, 21. XY; negative CMV and toxoplasma PCR in LA; negative TORCH serology. An extended genetic study revealed a pathogenic missense variant. This variant was identified in apparent homozygosis in the *PSAT1 gene*, associated with NLS-2 by autosomal recessive inheritance. Through a segregation study of the parents, it was observed that both were carriers of the *PSAT1 gene*. At this point, the pregnant woman was informed of the known prognosis for this syndrome to date and the possibility of continuing with the pregnancy to the benefit of the Organic Law of Sexual and Reproductive Health (SRH). Finally, with the help of the Obstetrics team and psychological support, the patient opted for legal termination of pregnancy.

Recently, this couple has become pregnant again and despite having been genetically counseled, they have had a bad outcome. The new pregnancy had a normal first and second-trimester ultrasound as well as a low-risk first-trimester screening for Down, Edward’s and Patau syndromes. Female sex. During the first-trimester, a chorionic biopsy was performed because no preimplantation genetic diagnosis (PGD) was performed. QF-PCR and CGH-arrays were normal. An extended genetic study revealed a non-pathogenic missense variant. This variant was identified in apparent heterozygosis in the *PSAT1 gene*, which theoretically does not imply disease. The fetus is a non-symptomatic carrier. However, at week 25 the patient was consulted due to decreased fetal movements, confirming the antepartum death of the fetus. They did not want further studies and they are currently being advised by the Perinatal Bereavement Unit of our center.

## 4. Discussion

As discussed in the previous clinical cases, arrays-CGH and QF-PCR were performed with normal results, respectively. Subsequently, the exome was studied by massive sequencing, detecting the homozygous variant of the missense type in the *PSAT1 gene* in the first fetus, classified as the pathogenic according to the guidelines of the American College of Medical Genetics and Genomics. The NM_058179:c/296C>T variant identified in the *PSAT1 gene* results in the substitution of an alanine for valine amino acid at position 99 of the protein (p(Ala99Val)) on chromosome 9. Polyphen2, LRT, MutationTaster, MutationAssesor, FATHMM, MetaSVM and MetaLR give a deleterious effect of the variant detected on the function or structure of the protein encoded by the *PSAT1 gene*, while the SIFT predictor gives it a tolerable effect. The variant is found in the Genome Aggregation Database with an allelic frequency of 0.02% and is registered in the dbSNP database in fetuses affected by Neu Laxova 2 syndrome, described as pathogenic in the ClinVar database [5,8]. There were no additional regions of homozygosity identified and no other variants of interest in this region. This suggests the parents likely share a distant common ancestor even if they do not know it. However, this variant in heterozygosis does not seem to be involved with the disease, although there are fewer reported data on this spectrum. Our findings place NLS at the severe end of the spectrum of serine-deficiency disorders. It is important to note that in the cohort of 12 families studied, Acuña-Hidalgo et al. did not identify any mutations in the three genes in two families, so other genes are thought to be involved in NLS. Therefore, the molecular mystery of NLS is far from being completely elucidated. Other molecular studies should follow to improve our understanding of this deadly metabolic disorder [7].

The *PSAT1 gene* has been described in the scientific literature as the cause, among other phenotypes, of Neu Laxova 2 syndrome, with an autosomal recessive model of inheritance. This disease is characterized by severe IUGR, severe microcephaly, severe CNS defects, such as ACC and hypoplastic cerebellum and brainstem, severe ichthyosis, and facial dysmorphism. An ultrasound at 19 or 20 weeks of gestation may show polyhydramnios, intrauterine growth retardation, hypoechoic skeletal structures, microcephaly, bulging eyes, retrognathia, and hypomobility with flexion deformities, so we can suspect this pathology [5,9].

As the result of these findings, we wanted to review all the cases published to date, representing the most important clinical and ultrasound features in the following table (Table 1). Of the 88 reported cases of NLS to date from genetics laboratories around the world [5], 81 have been published in the literature in different articles over the years (but the vast majority of them are cases of postnatal diagnosis, cases of prenatal diagnosis are fewer), which are the ones we have analyzed in this review [4,9,10,11,12,13,14,15,16,17,18,19,20]. In most cases, the findings have been studied postmortem by necropsy. With this table, we wanted to collect findings that can also be interpreted by ultrasound for prenatal management. For this reason, striking signs, such as ichthyosis, have not been included in the table.

Sex does not seem to be a predisposing factor. Diagnosis usually occurs during the second-trimester ultrasound through indirect signs. Although, several cases of diagnosis in the first-trimester through increased nuchal translucency (NT) have been identified [8]. For this reason, we want to emphasize the importance of ultrasound in all its stages. The karyotype was normal in most cases. It was not until 2014 when the extended studies of the human exome began, revealing the three genetic anomalies: *PHGDH*, *PSAT1* and *PSPH genes* that we explained before [7]. In our sibling fetuses, the mutated gene found was *PSAT1*, homozygous in case 1 and heterozygous in case 2. Regarding consanguinity, 45% of the cases were confirmed to be relatives, the rest did not confirm it but the possibility of distant ancestors cannot be ruled out.

The main manifestation, which is generally the first ultrasound finding in the second-trimester ultrasound, is IUGR. It is usually severe, early and determines a poor prognosis [14]. IUGR was reported in most cases (87% of cases) [4,5,6,7,8,9,10,11,12,13,14,15,16,17,18,19,20]. Skin abnormalities associated with NLS are typical, such as subcutaneous edema and ichthyosis. Ichthyosis, although characteristic, is not easy to diagnose intrauterine [11]. Subcutaneous edema is a frequent sign with variable intensity (73% of cases) [4,5,6,7,8,9,10,11,12,13,14,15,16,17,18,19,20]. It is often important in the extremities giving the appearance of “inflated gloves” and in its most severe form can be seen as generalized hydrops fetalis [12,13]. In our case one subcutaneous edema was present, but not hydrops.

Severe CNS malformations have been consistently seen in patients with NLS on ultrasound and/or neuropathological examination and have been considered “mandatory” manifestations of NLS. They are dominated by severe microcephaly (85% of cases) with other associated anomalies, such as cerebellar hypoplasia (36% of cases), lissencephaly (45% of cases), CC agenesis or hypoplasia (36% of cases), ventriculomegaly (17% of cases) and other CNS anomalies (<10% of cases) [4,5,6,7,8,9,10,11,12,13,14,15,16,17,18,19,20]. These include vermis agenesis, neural tube closure defects type of anencephaly or spina bifida, or Arnold–Chiari type II malformations [21]. In our case 1, the CNS alterations were dominated by CC agenesis and microcephaly. Hypoplastic cerebellum, lissencephaly and ventriculomegaly were not found. The post-mortem study was not carried out, so we lack the data.

Craniofacial dysmorphism is very characteristic and considered quite specific to NLS. This comprises severe microcephaly, sunken forehead, proptosis with rudimentary eyelids that give the impression of an “absence of eyelids”, hypertelorism, micrognathia, flattened nose, rounded and open mouth with thick lips and misshapen ears that are often low inserted [12,15,17]. Unfortunately, not all of these signs can be diagnosed prenatally by ultrasound. About the signs that can be seen sonographically: 68% of the cases presented micrognathia, 56% ocular proptosis, 79% flattened nose, 81% flattened forehead and 49% hypertelorism [4,5,6,7,8,9,10,11,12,13,14,15,16,17,18,19,20]. In our case 1, the only sign not present was hypertelorism.

Among limb anomalies, arthrogryposis stands out as one of the most constant signs of NLS (80% of cases) and is usually suggested on prenatal ultrasound [4,5,6,7,8,9,10,11,12,13,14,15,16,17,18,19,20]. It is part of the so-called hypokinesia sequence, fetal or cerebro-arthro-digital, secondary to severe deterioration of the CNS and ichthyosis [18,21]. It is associated with muscle hypotrophy and bone abnormalities. The latter, linked to a defect in bone modeling, are characterized by kyphoscoliosis [21]. In our case 1, flexion contractures of the wrist joints were found. No syndactyly was observed, present in 48% of cases. Other abnormalities frequently associated with NLS, including pulmonary and genital hypoplasia, were also not observed. Finally, some anomalies are rarer, such as cardiopathies (atrial or ventricular septal defect), renal malformations (hydronephrosis, renal hypoplasia, or agenesis), or cleft lip (<10% of cases). None of them were observed in our case 1. In addition, polyhydramnios is a frequent sonographic sign (31% of cases) [4,5,6,7,8,9,10,11,12,13,14,15,16,17,18,19,20] that was not present in our case 1.

The differential diagnosis of the NLS arises prenatally, mainly with the other syndromes associated with a sequence of fetal hypokinesia or akinesia. It should include cerebro-ocular-facial-skeletal (COFS) syndrome, Walker–Warburg syndrome, Pena–Shokeir syndrome type I, cerebro-arthrodigital syndrome, Smith–Lemli–Opitz syndrome and Miller–Dieker syndrome (Table 2) [3].

Serine supplementation of affected embryos appears to be an attractive treatment option for the treatment of NLS or at least to reduce the severity of the development deficit. This therapeutic approach faces the difficulty of establishing a molecular diagnosis of the disease at the stage of embryonic development. This early prenatal diagnosis is theoretically possible in families with a high risk of recurrence and where the mutation was previously identified in the index case [22].

## 5. Conclusions

NLS is a lethal entity characterized by huge phenotypic and genetic heterogeneity. Only eighty-eight cases have been reported to date. In the diagnosis, the collaboration of the Genetics and Prenatal Diagnosis Services is essential. The disorder is characterized by severe IUGR, microcephaly, ichthyosis, short neck, limb deformities, hypoplastic lungs, subcutaneous edema, facial dysmorphism (marked proptosis, micrognathia, hypertelorism, flattened nose and malformed ears) and CNS anomalies. It can be induced by a mutation to one of three genes involved in de novo serine synthesis: *PHGDH*, *PSAT1*, and *PSPH*. Apparently, heterozygosis in the *PSAT1 gene* theoretically does not imply disease. There are also more genes involved yet to be elucidated [4]. In our case, with a homozygosis mutation identified in the *PSAT1 gene*, preimplantation genetic diagnosis or prenatal genetic diagnosis is a good option in future pregnancies. Public awareness in the form of genetic counseling and the risks associated with consanguinity should be emphasized to reduce the incidence of NLS [23].

In general, the prognosis is poor. However, the variety in the phenotypic spectrum has allowed the expansion of prognostic ranges. In addition, serine supplementation of affected embryos appears to be an attractive future treatment option for patients affected by NLS. Although more studies should be carried out that can demonstrate the safety and effectiveness intrauterine and after birth [23].

## Figures and Tables

**Table 1:** Review of NLS cases published to date, representing the most important clinical and ultrasound features.

	Literature (83 Cases)	Case 1	Case 2
**Sex**	57% Female; 33% Males	Male	Female
**Age (weeks)**	12–41	21	13
**Consanguinity**	45%	No	
**Karyotype**	Normal 73%	Normal	
**Gene**	*PHGDH, PSAT1 and PSPH*	*PSAT1 in homozygosis*	*PSAT1 in heterozygosis*
**IUGR**	87%	Yes	No
**Hydrops or subcutaneous edema**	73%	Yes	No
**CNS malformations**			
*Microcephaly*	85%	Yes	No
*Hypoplastic cerebellum*	36%	No	No
*Lissencephaly*	45%	No	No
*Agenesis/hypoplasia of CC*	36%	Yes	No
*Ventriculomegaly*	17%	No	No
**Craniofacial dysmorphism**			
*Micrognathia*	68%	Yes	No
*Ocular proptosis*	56%	Yes	No
*Flattened nose*	79%	Yes	No
*Flattened forehead*	81%	Yes	No
*Hypertelorism*	49%	No	No
**Limb abnormalities**			
*Arthrogryposis*	80%	Yes	No
*Syndactyly*	48%	No	No
**Pulmonary hypoplasia**	39%	No	No
**Polyhydramnios**	31%	No	No
**Cardiopathy**	6%	No	No
**Others**	<5%	No	No

**Table 2:** Differential diagnosis of the NLS [3].

**COFS**	Craniofacial malformations, ocular abnormalities, musculoskeletal defects, malformations and progressive degenerative changes of the brain and spinal cord.
**Walker-Warburg syndrome**	Severe congenital oculo-cerebral abnormalities, including lissencephaly and ventriculomegaly.
**Cerebro-arthrodigital sd**	Arthromyodysplasia, dyscephaly, sacral agenesis, and hypoplastic digitis.
**Pena-Shokeir syndrome type I**	Abnormal fetal movement profile, craniofacial malformations, pulmonary hypoplasia, IUGR.
**Smith-Lemli-Opitz syndrome**	Facial anomalies, mental retardation, pre- and postnatal growth disorder and abnormalities in the external genitalia.
**Miller-Dieker syndrome**	It is a variety of lissencephaly, where the brain presents with few or no convolutions.

## Data Availability

All data generated or analyzed during this study are included in this published article.
